# Guess what? Only correct choices forge immediate stimulus–response bindings in guessing scenarios

**DOI:** 10.3758/s13414-024-02950-2

**Published:** 2024-09-17

**Authors:** Anna Foerster, Viola Mocke, Birte Moeller, Roland Pfister

**Affiliations:** 1 Trier University, Trier, Germany; 2 University of Würzburg, Würzburg, Germany

**Keywords:** Action control, Binding and retrieval, Rules, Error processing, Instructions

## Abstract

A central mechanism of human action control is the prompt binding between actions and the stimuli provoking them. Perceiving the same stimuli again retrieves any bound responses, facilitating their execution. An open question is whether such binding and retrieval only emerges when stimulus–response rules are known upon taking action or also when agents are forced to guess and receive feedback about whether they were successful or not afterward. In two experiments, we tested the hypothesis that knowing rules before responding would boost binding between stimuli and responses during action-taking relative to guessing situations. Second, we assessed whether the content of the feedback matters for binding in that agents might use feedback to build correct stimulus–response bindings even for wrong guesses. We used a sequential prime-probe design to induce stimulus–response binding for prime responses that were either rule-based or guesses, and to measure retrieval of these bindings in response times and errors in the probe. Results indicate that binding and retrieval emerge for successful but not for wrong guesses. Binding effects for correct guesses were consistently small in effect size, suggesting that pre-established stimulus–response bindings from instructed rules might indeed boost binding when taking action.

## Introduction

Actions become easier the more experience one gathers with their specific situational demands. That is, because even for a single action, features of the action and of the situation in which it takes place are bound together. This way, an action can be retrieved upon re-encountering familiar situational features (e.g., Frings et al., 2020, 2024). Binding and retrieval of perceptual and behavioral features through experience therefore offer short-cuts for selecting actions easily across similar situations. At the same time, actions also become easier if they are guided by specific instructions on what to do. This works through binding and retrieval of instructed stimulus–response rules, even without prior experience (e.g., Wenke et al., 2007). But how do these two factors interact? Do rules affect how experience builds up? We approached these questions by studying binding and retrieval for responses that were either guesses in unfamiliar situations or informed responses based on previously established stimulus–response rules.

### Being right, being wrong

No matter whether agents know exactly how to act in a specific situation or whether they must guess, their selected action can end up being correct or wrong. In situations where the agent knows an appropriate rule and intends to apply this rule, the execution of the plan can still go wrong from time to time (Reason, 1995). Many researchers studied the consequences of these commission errors for action control in the last decades. Some critical implications of these investigations are that commission errors are registered and cancelled quickly (e.g., Foerster et al., 2022b; Hochman et al., 2017; Rabbitt, 1978; Roger et al., 2014), with frequent and prompt attempts to correct them (e.g., Crump & Logan, 2013; Rabbitt, 1966). Error commission seems to occupy information processing even after the execution of the erroneous action ended, as it elicits a longer lasting shift toward more cautious action-taking, evident in a slow-down of actions following errors (e.g., Jentzsch & Dudschig, 2009; Steinhauser et al., 2017).

Sometimes, however, agents cannot know the right course of action, and being right or wrong depends on luck. In gambling situations, where the rules of the game are clear but outcomes are uncertain, agents shift toward more impulsive, that is, faster, action initiation after a loss than after a win (e.g., Eben et al., 2020; Forder & Dyson, 2016; Verbruggen et al., 2017). This shift also occurs when losing a gamble against an unpredictable opponent. However, losing against an, in principle, exploitable opponent induced slowing in subsequent responses as observed for commission errors (Dyson et al., 2018). These findings are in line with a recent study that directly compared commission errors, for which rules were known and applicable, with different types of guesses (Eben et al., 2023). For guesses, participants had to categorize the color of a stimulus as light or dark gray when in fact, the stimulus always had the same shade of gray. The researchers instructed one group of participants to categorize the color even though it would be difficult and they instructed the other group of participants to select a response although it was impossible for the human eye to see the difference in color. So, either a rule was instructed but in fact not applicable in the task, or participants knew right from the beginning that there was no rule and they had to guess the correct response to a presented stimulus. Participants slowed down after commission errors for known and applicable rules but sped up after wrong guesses, with and without an instructed rule. As such, behavior seems to adapt flexibly depending on the controllability of being wrong in the situation.

Crucially, being wrong can also inform agents about what would be a correct action when re-encountering a similar situation. This has been documented in studies on trial-and-error learning, in which participants guessed a correct response and then received feedback about whether the chosen action was right or wrong (e.g., Behrens et al., 2007; Rescorla & Wagner, 1972). Even though trial-and-error learning can unfold quickly for a single behavioral instance, it is usually related to specific learning strategies to encode stimulus–response rules or response-outcome contingencies (Lee et al., 2015; Mohr et al., 2018). Here we extend this line of work to binding and retrieval mechanisms operating at the moment a guess is made and feedback about its correctness is received.

### Shortcuts to action selection via binding and retrieval

A plethora of studies found converging evidence with regard to the notion that whenever we act, or also when we merely plan an action or receive instructions about rules, there is binding between features of the stimulus and of the response and between features of the response and its effects (e.g., Frings et al., 2020, 2024; Henson et al., 2014; Hommel et al., 2001; Liefooghe et al., 2012; Theeuwes et al., 2015). Crucially, encountering the same stimulus or effect after such a binding instance again automatically retrieves features of the bound response. If this response is again appropriate, its execution is easier. If it is inappropriate, it hampers execution of other responses. Such short-cuts to action control also seem to emerge when agents commit errors in rule-based situations (Foerster et al., 2023, 2021, 2022a; Parmar et al., 2022). Crucially, not the executed erroneous response but the omitted correct response forms a binding with the acted-upon stimulus, whereas the executed erroneous response enters a binding with the effects it produced. As such, binding appears to be flexible and adaptive in the face of error commission because it considers existing task rules but also experience-based contingencies.

### The present study

**Fig. 1:**
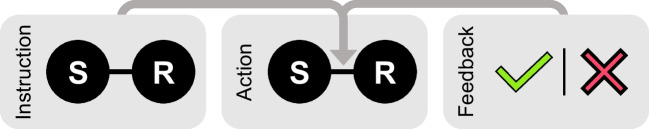
Illustration of the central research questions. Bindings from instructed stimulus–response rules might feed into binding of the features of stimulus and response during action-taking. If instructions are not available, feedback might allow for an adjustment of binding after guessing, depending on whether the guess was correct or wrong

If existing task rules shape the binding of stimuli and responses after an error, these rules might also generally feed into bindings during action planning (see Fig. 1). Many empirical studies consistently point to binding of stimulus–response rules as already occurring during their verbal instruction, and retrieval of that binding upon first stimulus presentation and action-taking (e.g., Cohen-Kdoshay & Meiran, 2009; Pfeuffer et al., 2017; Wenke et al., 2007). As such, instructed rules and experienced action episodes share a similar binding-based structure, and the current study set out to investigate their interplay.

**Fig. 2:**
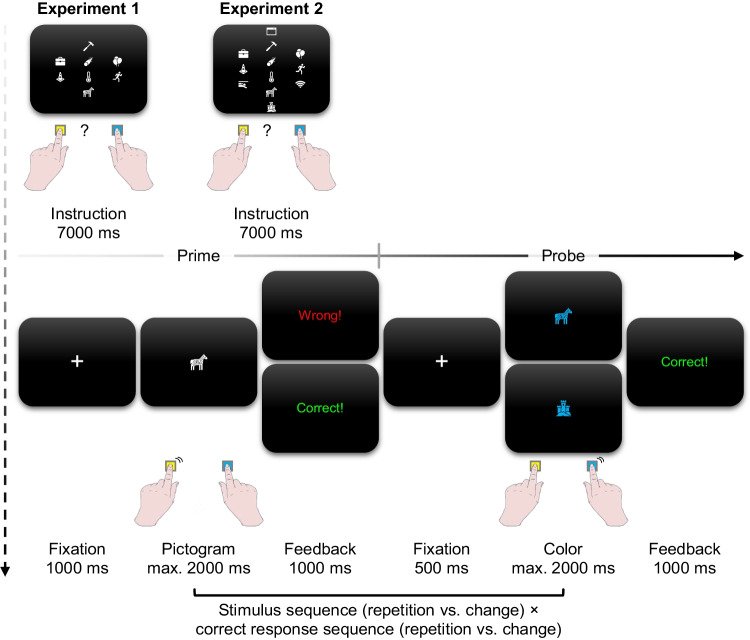
Exemplary instruction of a mini block with its first out of eight trials in Experiments 1 and 2. The instructions included either eight (Experiment 1) or 12 pictograms (Experiment 2). Half of these pictograms required a rule-based left or right response, indicated by their position on the screen. For the other half of the pictograms, presented in the middle of the screen, the correct response had to be guessed. In the prime part of the trial, one of these pictograms appeared and required either a rule-based response or a guess with the left or right key (here: zebra from the guessing category). For guesses, participants received 50% ”correct” and 50% ”wrong” feedback, irrespective of the response. For rule-based pictograms, the feedback reflected adherence to the instructed rules. In the probe part of the trial, participants had to ignore the identity of the pictogram and respond to its color. They received feedback about the accuracy of each probe response. The pictogram repeated or changed from prime to probe as did the correct response. For stimulus changes, in Experiment 1, probe pictograms had not been part of the instructed pictogram set and were therefore novel (here: castle). In Experiment 2, they had been part of the instructed pictogram set and they belonged to the same instruction category as the prime pictogram (both rule-based left, rule-based right or guess, here: castle)

In both experiments, we presented participants with a collection of pictograms for an upcoming block of trials to instruct them how to respond to each of them, based on rules or guesses (see Fig. 2). Some pictograms were allocated to the left and right side of the screen specifying the correct response via a rule. Other pictograms appeared centrally, indicating that participants had to guess the correct response. Participants were told to memorize the assignment of all pictograms to the two specific responses or to guessing.

Taken together, like in one of the previously described studies (Eben et al., 2023), participants could apply a rule to classify one half of a set of stimuli, but could not apply a rule and instead had to guess the correct response for the other half of stimuli. A big methodological difference is that we prepared a rather large set of different stimuli here. This way, participants provided only one rule-based response or one guess for each pictogram. This procedure prevents contingency learning and therefore allowed us to isolate short-term binding and retrieval.

We measured binding and retrieval for these rule-based or guessed responses to the pictograms via a sequential prime-probe paradigm. In the prime, participants responded to the identity of a pictogram with a left or right keypress in line with the previously provided instruction (left vs. right vs. guess). Half of the guesses were fed back as being correct and the other half as being false. We assumed that binding of the identity of the pictogram and the response would take place in the prime. In the probe, participants categorized the color of a pictogram, while they had to ignore its irrelevant identity. The identity of the pictogram in the probe was the same as in the prime or novel (stimulus repetition vs. stimulus change). We assumed that a repetition of the identity of the pictogram from prime to probe would retrieve the bound response from the prime. To assess whether such binding and retrieval happens, we further manipulated the sequence of the *correct* responses from prime to probe. For correct rule-based responses and correct guesses in the prime, a repetition of the correct response meant that the executed prime response was also correct in the probe. For wrong rule-based responses and wrong guesses instead, a repetition of the correct response entailed that the correct, but not executed, prime response was correct in the probe and that the executed, wrong response was not appropriate in the probe. The interaction of stimulus sequence (repetition vs. change) and correct response sequence (repetition vs. change) in probe response times and probe error rates informs about whether a response had been bound to the stimulus in the prime and is retrieved in the probe. In particular, the direction of the effect indicates whether the correct response or the erroneous response was subject to binding and retrieval for all three prime types.

We hypothesized that access to existing stimulus–response rules would promote binding and retrieval when these rules are put into action compared to guessing situations where agents must act without knowing the correct mapping. Therefore, we compared sequential stimulus–response effects between prime response types (i.e., rule-based vs. correct guess vs. wrong guess). For rule-based prime responses, we expected sequential stimulus–response effects with a larger benefit (i.e., faster responses and fewer errors) when repeating over changing the correct response when stimuli repeated relative to when stimuli changed. This effect should be in the same direction but reduced both for correct and wrong guesses if the accessibility of the stimulus–response rule before responding boosts binding.

The second aim of this study was to establish whether guessed responses are bound to and later also retrieved by the response-evoking stimuli, and if so, whether binding and retrieval depend on the correctness of the guesses. Specifically, making an incorrect guess might result in the stimulus being bound to the correct instead of the executed incorrect response as has been observed for commission errors in rule-based situations (Foerster et al., 2023, 2022a). However, we do not know whether such an adjustment is even possible through feedback after the execution of a response that has been prepared without a rule at hand. If this adjustment occurs sometimes but unreliably, binding and retrieval of the correct response should be evident for both guesses, but it should be weaker for wrong compared to correct ones, resulting in sequential stimulus–response effects in the direction as described above but smaller effects for wrong guesses. If an adjustment is not possible at all, there should even be binding and retrieval of the executed wrong response to the stimulus instead, resulting in reversed stimulus–response effects (i.e., a larger benefit of changing the correct response when stimuli repeated relative to when stimuli changed).

## Experiment 1

### Method

#### Participants

Sequential interaction effects between relevant stimuli and correct responses in correct action episodes with proper instructions about the task rules amounted to *d*_*z*_ ≥ 1.41 in response times (RTs) in recent studies from our lab (Foerster et al., 2023, 2022a). The modulation of these sequential stimulus–response effects through the accuracy of a guessed response amounted to *d*_*z*_ = 0.41 in RTs in an unpublished study from our lab (n = 32, https://osf.io/mcys5/) with small sequential stimulus–response effects after both correct guesses, *d*_*z*_ = 0.35, and wrong guesses, *d*_*z*_ = -0.28 (Foerster & Pfister, 2022).

Considering that the reported sequential stimulus–response effects were much larger in the past for rule-based responses than for both correct and wrong guesses, we estimated that a modulation of binding and retrieval effects by prime response type could be of medium size. In contrast to the unpublished study, the current study introduced rule-based responses alongside guesses, requiring the processing of the identity (not only the onset) of the pictograms and their relation to one or no response (as learned during instructions). As such, we expected larger sequential stimulus–response effects for guesses in the current than in the unpublished study. A medium-sized effect therefore seemed to be a reasonable basis for the power analysis. A sample of 44 participants has a power of 90% to detect a medium-sized effect of *d*_*z*_ = 0.50^1^ in a two-tailed paired-samples test with an alpha of 5% (calculated with the power.t.test function in R version 4.0.3; R Core Team, 2023).

We planned to conduct studies with eight participants, respectively, until one study fulfilled the criterion that at least 75% of the participants could be included in the data analyses (see *Data treatment* for exclusion criteria). As the first study fulfilled that goal, we filled up the sample according to the power analysis above. In the end, we had to collect 67 participants for 44 analyzable datasets (35 female, nine male, none non-binary; 40 right-handed, three left-handed, one ambidextrous; age: *M* = 27 years, *SD* = 6 years). All participants provided informed consent.

#### Apparatus and stimuli

The study was conducted in a laboratory at the University of Würzburg. Participants responded with their left and right index fingers on the keys *F* and *J* on a QWERTZ keyboard. In each trial, participants had two tasks. They first responded to the identity of white pictograms in the prime and then they responded to the color (blue vs. yellow) of a pictogram in the probe (see Fig. 2). We used 588 pictograms (from the Microsoft® Office library) in the experiment and assigned them randomly to one of the two possible responses (i.e., rule-based responses), to guesses in the prime, or to the role of novel pictograms in the probe. That is, an individual pictogram appeared only in a single trial of the whole experiment, and in this trial, it appeared only in the prime, only in the probe, or in both prime and probe. We counterbalanced the assignment of colors to responses in the probe across participants.

#### Procedure

The experiment was structured into nine blocks. The first block was a practice block and included one mini block. The other eight experimental blocks featured six mini blocks each. The experiment offered self-paced breaks after each experimental block with a reminder to conduct both tasks as quickly and accurately as possible. Each mini block featured eight trials. At the beginning of each mini block, participants had to memorize the assignment of eight novel white pictograms to the two response keys: Two pictograms depicted on the left side of the screen mapped to the left key, the two on the right side of the screen mapped to the right key (i.e., four rule-based pictograms), and the participants had to guess the correct response for the remaining four centrally presented pictograms. This instruction stayed on-screen for 7,000 ms.

We instructed participants at the beginning of the experiment to memorize the assignment of all pictograms to the two specific responses or to the guesses as quickly and as appropriately as possible. We further instructed them to respond as fast as possible with a left (right) keypress whenever the prime showed a pictogram that had appeared on the left (right) side of the screen during the instruction. We further instructed them that they should guess the correct response as quickly as possible whenever a pictogram appeared for which the correct response was unknown. We encouraged them to make a spontaneous decision based on their gut feeling, as if flipping a coin. We also advised them that they did not need to balance their guesses between the left and right responses.

After a long fixation for 1,000 ms, one of the eight pictograms appeared in white, and participants had to respond within 2,000 ms. Upon a keypress, visual feedback informed participants about the accuracy of each prime response for 1,000 ms: “Correct!” (German: “Korrekt!”) in green font color, “Wrong!” (German: “Falsch!”) for a false keypress or “Too slow!” (German: “Zu langsam!”) for response omissions in red font color. For rule-based responses, the feedback reflected what participants did. For guessed responses, we randomly provided correct and wrong feedback. To reduce predictability, we presented “Correct!” for 12 guesses and “Wrong!” for the other 12 guesses in a random order across the six mini blocks within each experimental block, so that the distribution of feedback types varied across mini blocks. If participants did not deliver a response in time, the feedback for omissions was presented instead.

After a short fixation of 500 ms, a colored pictogram appeared in the probe. The irrelevant pictogram was either the same as in the prime or a novel pictogram. We instructed participants in the beginning of the experiment to respond as quickly and accurately to the color while ignoring the pictogram. The color was selected depending on the sequence of correct responses from prime to probe (repetition vs. change) in the current trial. In the case of an erroneous rule-based prime response or an incorrect guess in the prime, the selection was still based on the actual correct but not executed prime response. We presented each of the four individual prime-probe sequences (2 stimulus sequences × 2 sequences of correct responses) six times for rule-based responses, three times for correct guesses and three times for wrong guesses, in a random order across six consecutive experimental mini blocks in a block to reduce predictability in the design.

### Results

#### Data treatment

We analyzed the data in R (R Core Team, 2023) and we used the R packages ez version 4.4–0 (Lawrence, 2016), schoRsch version 1.10 (Pfister & Janczyk, 2016), and tidyverse version 2.0.0 (Wickham et al., 2019).

The research assistant re-started the experiment after a technical failure in the middle of the experiment for one participant and right after the practice block for three participants because they had problems with conducting the task. However, these participants interacted with some of the pictograms multiple times, and we therefore had to exclude and replace their data although we had not anticipated these exclusions in the preregistration. All following exclusions and replacements were preregistered. From the remaining dataset, we excluded the first mini block as practice. We excluded and replaced two participants who responded correctly in less than 75% of the rule-based primes. All remaining participants responded correctly in at least 75% of the probes. We had to exclude and replace 17 participants because they preferred one of the response keys in more than 70% of the guessed prime responses. We excluded trials with an error in the probe of the preceding trial from analysis (5.1% commission errors and 0.2% omission errors). We then excluded trials with an early response before the prime during fixation (< 0.1%), trials with a commission error in rule-based primes (2.4%), trials with an omission of the prime response (0.3%) or with a response between prime and probe (0.3%). For the analysis of probe error rates, we excluded probe omission errors (0.2%). For the analysis of probe RTs, we further excluded commission errors in the probe (5.1%) and then outlier trials where RTs of the probe deviated more than 2.5 standard deviations from their respective cell mean (2.1%). All remaining participants delivered at least ten observations in all experimental cells after these exclusions and could be included in the analyses.

#### Data analyses

**Fig. 3:**
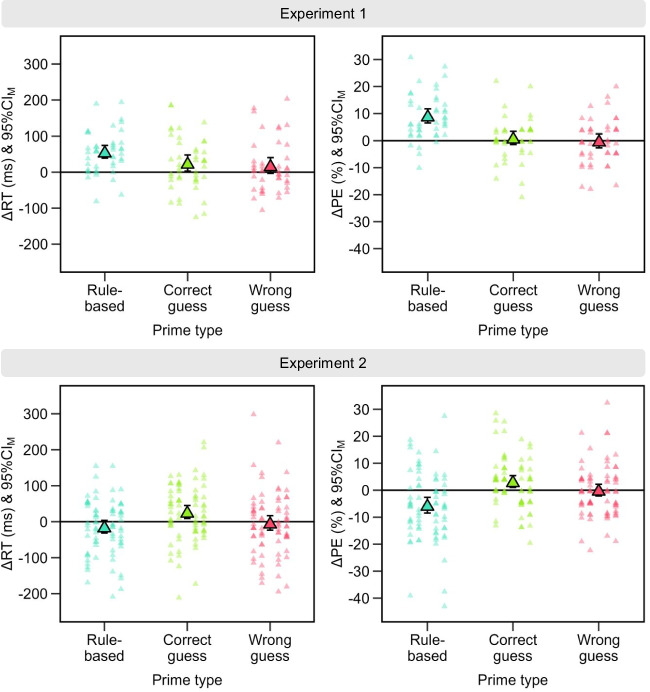
Main results. Sequential stimulus–response effects for the response time (ΔRT, left) and for the percentage error (ΔPE, right) in Experiment 1 (top) and in Experiment 2 (bottom) for the three prime types, that is, rule-based, correct guess, and wrong guess. Positive values point to retrieval of the former correct response upon stimulus repetition. The big triangles show the condition means with their 95% confidence interval of the mean (CI_M_). The smaller triangles show individual values of each participant

**Fig. 4:**
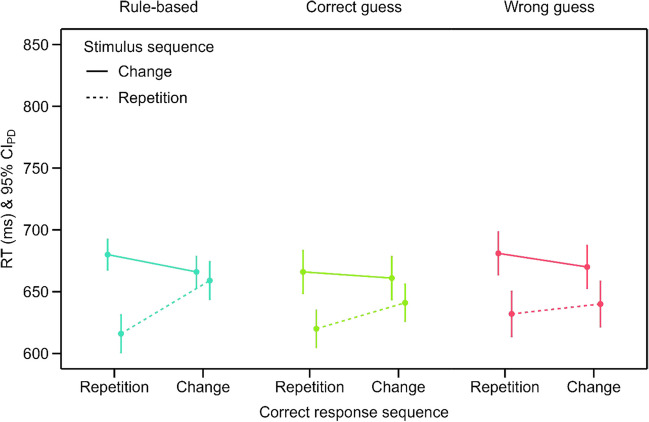
Detailed results for the response time (RT) in Experiment 1. Means are depicted as a function of stimulus sequence and correct response sequence for the three prime types, that is, rule-based, correct guess and wrong guess. Error bars represent the 95% confidence interval of the paired differences between correct response sequences (CI_PD_), computed separately for stimulus sequences and prime types

**Fig. 5:**
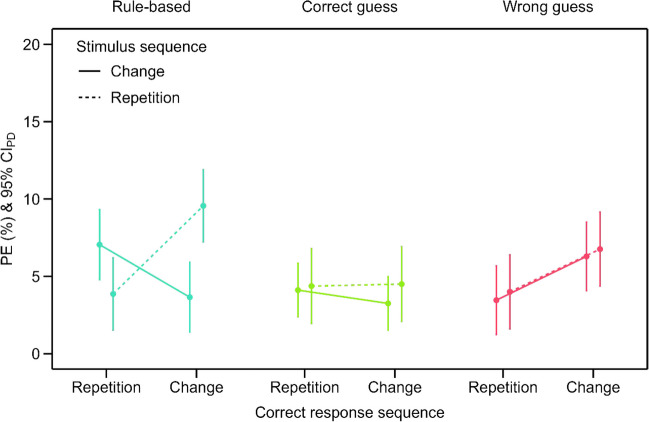
Detailed results for the percentage error (PE) in Experiment 1. Means are depicted as a function of stimulus sequence and correct response sequence for the three prime types, that is, rule-based, correct guess and wrong guess. Error bars represent the 95% confidence interval of the paired differences between correct response sequences (CI_PD_), computed separately for stimulus sequences and prime types

We analyzed RTs of correct probe responses in a 2 × 2 × 3 analysis of variance (ANOVA) with the within-subject factors prime type (rule-based vs. correct guess vs. wrong guess) × stimulus sequence (repetition vs. change) × correct response sequence (repetition vs. change). We scrutinized significant two-way interactions in two-tailed paired-samples *t*-tests. If the three-way interaction was significant, we computed sequential stimulus–response effects (Δ = [stimulus change and correct response repetition – stimulus repetition and correct response repetition] – [stimulus change and correct response change – stimulus repetition and correct response change]) and assessed (1) whether these differed between the prime types in two-tailed paired-samples *t*-tests and (2) whether these were different from 0 in three two-tailed one-sample *t*-tests.^2^ We analyzed percentages of commission errors in the probe as described above for RTs to address potential speed-accuracy trade-offs. Figure 3 shows key results for the RT and error analyses. In the Appendix, we present absolute RTs and percentage errors for each design cell in Figs. 4 and 5 and also in Tables 1 and 2.

#### Response times

Stimulus repetitions were faster than stimulus changes (Fig. 4 in the Appendix), *F*(1, 43) = 71.70, *p* < 0.001, η_p_^2^ = 0.63, and correct response repetitions were faster than correct response changes, *F*(1, 43) = 4.16, *p* = 0.048, η_p_^2^ = 0.09. The main effect of prime type was not significant, *F*(2, 86) = 2.16, *p* = 0.134, η_p_^2^ = 0.05 (ε = 0.78). Prime type did not interact with stimulus sequence, *F* < 1, or correct response sequence, *F*(2, 86) = 1.57, *p* = 0.219, η_p_^2^ = 0.04 (ε = 0.70). The interactions between stimulus sequence and correct response sequence (see Fig. 3), *F*(1, 43) = 32.57, *p* < 0.001, η_p_^2^ = 0.43, and between all three factors were significant, *F*(2, 86) = 3.82, *p* = 0.035, η_p_^2^ = 0.08 (ε = 0.81).

In line with binding and retrieval of the stimulus and the correct response, correct response repetitions were faster than correct response changes for stimulus repetitions, *t*(43) = 5.25, *p* < 0.001, *d*_*z*_ = 0.79, whereas there was a reversed effect for stimulus changes, *t*(43) = -2.19, *p* = 0.034, *d*_*z*_ = -0.33. This sequential stimulus–response effect was larger for rule-based responses compared to correct guesses, *t*(43) = 2.57, *p* = 0.014, *d*_*z*_ = 0.39, and compared to wrong guesses, *t*(43) = 2.83, *p* = 0.007, *d*_*z*_ = 0.43, whereas it did not differ between the two guessing types, |*t*|< 1. It was only significant for rule-based responses, *t*(43) = 6.47, *p* < 0.001, *d*_*z*_ = 0.98, and correct guesses, *t*(43) = 2.29, *p* = 0.027, *d*_*z*_ = 0.34, but not for wrong guesses, *t*(43) = 1.72, *p* = 0.093, *d*_*z*_ = 0.26.

#### Percentage of commission errors

The percentage of commission errors differed between prime types (see Fig. 5 in the Appendix), *F*(2, 86) = 8.60, *p* < 0.001, η_p_^2^ = 0.17, with fewest errors after correct guesses, followed by wrong guesses and most errors after rule-based primes. The main effect of stimulus sequence was not significant, *F*(1, 43) = 3.80, *p* = 0.058, η_p_^2^ = 0.08. Correct response changes were more frequently erroneous than correct response repetitions, *F*(1, 43) = 5.57, *p* = 0.023, η_p_^2^ = 0.11. The two-way interactions between prime type and stimulus sequence, *F* < 1, and between prime type and correct response sequence, *F*(2, 86) = 2.87, *p* = 0.082, η_p_^2^ = 0.06 (ε = 0.71), were not significant. The two-way interaction between stimulus and correct response sequence (see Fig. 3), *F*(1, 43) = 29.93, *p* < 0.001, η_p_^2^ = 0.41, as well as the three-way interaction, *F*(2, 86) = 13.89, *p* < 0.001, η_p_^2^ = 0.24, were significant.

In line with binding and retrieval of the stimulus and the correct response, there were more errors if the prime and probe required the same correct response than if the correct response changed for stimulus repetitions, *t*(43) = 4.48, *p* < 0.001, *d*_*z*_ = 0.68, whereas the percentage of errors did not differ between correct response sequences for stimulus changes, |*t*|< 1. The sequential stimulus–response effect was larger for rule-based primes than for both correct guesses, *t*(43) = 4.89, *p* < 0.001, *d*_*z*_ = 0.74, and wrong guesses, *t*(43) = 4.57, *p* < 0.001, *d*_*z*_ = 0.69, whereas it did not differ between guessing types, |*t*|< 1. It was only significant for rule-based primes, *t*(43) = 7.02, *p* < 0.001, *d*_*z*_ = 1.06, not for correct guesses or wrong guesses, |*t*s|< 1.

#### Exploratory analyses

To explore whether rule-based responses and guesses differed from each other, we analyzed RTs of the prime in a two-tailed paired-samples *t*-test. We applied all selection criteria to the trials as in the RT analysis of the probes described above. In addition, we excluded outlier trials where RTs of the prime deviated more than 2.5 standard deviations from their respective cell mean (2.7%). All participants delivered at least ten observations in the two experimental cells after these exclusions and were included in the analyses. RTs in the prime were shorter for rule-based responses (*M* = 663 ms) than for guesses (*M* = 777 ms), *t*(43) = 5.98, *p* < 0.001, *d*_*z*_ = 0.90.

### Discussion

The modulation of correct response sequence by stimulus sequence both in RTs and errors points to the retrieval of bindings between acted-upon stimuli and correct responses. That is, the repetition of the pictogram from prime to probe seems to have retrieved the correct prime response. This retrieval was beneficial if the correct response from the prime was again correct in the probe but hampered responding if the correct response changed from prime to probe. This sequential stimulus–response effect was larger for instructed responses than for guesses, pointing to an impact of existing rules on binding and retrieval effects. Although sequential stimulus–response effects did not differ between correct and wrong guesses, they emerged significantly only for correct guesses in RTs but not for wrong guesses.

After conducting the first study, we noticed that stronger sequential stimulus–response effects for rule-based responses than guesses do not necessarily reflect a boost of binding and retrieval through rules because we introduced a confound in the paradigm. In rule-based trials, stimulus repetitions systematically differed from stimulus changes in terms of their access to a pictogram-response rule in the probe. In stimulus change trials, participants encountered a pictogram in the probe that had neither been part of the instructions nor had it been presented in the prime. In stimulus repetition trials, the irrelevant pictogram not only appeared in the preceding prime but it also got assigned a response rule in the preceding instruction. Thus, encountering a rule-based (now irrelevant) pictogram in the probe, might have retrieved the instructed rule-based response, which would be compatible with the required color response in correct response repetition trials, facilitating its execution, whereas the two responses would be incompatible in correct response change trials, hampering the execution of the correct probe response. As such, both retrieval of a response rule in the probe or retrieval of a binding from the prime episode could have been responsible for the sequential stimulus–response effects we observed for rule-based primes. In contrast, an instructed rule could not be retrieved from a pictogram in the probe after a guess in the prime, even if the identity of the pictogram repeated from prime to probe.

## Experiment 2

### Introduction

In the second experiment, we eliminated the confound from Experiment 1 to scrutinize whether binding and retrieval is indeed stronger for rule-based actions than for guesses, for correct than wrong guesses, and whether binding and retrieval emerges for all three types of actions. Crucially, we adapted the selection of stimuli in stimulus change trials: Probe pictograms in stimulus change trials also appeared during the instruction phase of the mini block in the same category as the preceding prime pictogram (rule-based left response, rule-based right response or guess; see Fig. 2). This selection of the pictograms in the probe allowed us to establish equivalent access to rule-based left or right responses from instructions in the probe across stimulus repetition and stimulus change trials, eliminating such a potential impact on sequential stimulus–response effects.^3^ A potential drawback of this procedure is that participants could already anticipate the instructed category of the irrelevant probe pictogram (i.e., rule-based left, rule-based right or guess) when encountering the prime pictogram, affecting probe processing. This was because now not only in stimulus repetition trials but also in stimulus change trials the response category was always repeated from prime to probe.

### Method

#### Participants

Differences in sequential stimulus–response effects between the three prime types were small (rule-based vs. correct guess: d_z_ = 0.39; rule-based vs. wrong guess: d_z_ = 0.43; correct vs. wrong guess: d_z_ = 0.06) in RTs in Experiment 1. Further, these effects were small to large within each prime type (rule-based: d_z_ = 0.98; correct guess: d_z_ = 0.34; wrong guess: d_z_ = 0.26).^4^

Detecting some of these very small effects would call for huge samples. Therefore, we aimed for a high power to detect effect sizes of d_z_ ≥ 0.34. A sample of 76 participants has a power of 90% to detect this effect size in a one-tailed, paired-samples *t*-test with an alpha level of 5% (calculated with the power.t.test function in R version 4.0.3; R Core Team, 2023). Even if the current experiment did not show any differences between rule-based and guessed responses, the sample size would still provide a power of 90% to detect sequential stimulus–response effects of d_z_ ≥ 0.38 across prime response types (stimulus sequence × correct response sequence in the ANOVA; inherently two-tailed, alpha = 5%).

As in Experiment 1, our first study fulfilled our goal of including at least 75% of the participants of the small sample (*n* = 8) in the analyses so that we proceeded with data collection. We had to collect 99 participants to have analyzable datasets of 76 participants (60 female, 14 male, two non-binary; 66 right-handed, ten left-handed; age: *M* = 25 years, *SD* = 5 years). All participants provided informed consent.

#### Apparatus, stimuli, and procedure

The study was conducted in laboratories of the University of Würzburg and Trier University. We used the same apparatus and stimuli as in Experiment 1. The procedure was very similar except for the following changes. We increased the number of pictograms in the instruction phase from eight to 12, with six rule-based pictograms distributed equally to the left and right responses and six pictograms for which the correct response had to be guessed.

One trial per mini block presented a rule-based left pictogram in the prime and repeated the same pictogram in the probe. Accordingly, two rule-based left pictograms remained in this mini block. These were used to create a stimulus change trial, so that one of the pictograms appeared in the prime and the other appeared in the probe. Analogously, we presented one stimulus repetition and one stimulus change trial with the rule-based right pictograms. Finally, two of the guessing pictograms were each presented in the prime and in the probe of the same trial (stimulus repetition), whereas the remaining four appeared either in the prime or in the probe (stimulus change). This selection procedure ensured that each instructed pictogram appeared in its associated mini block. However, to reduce predictability, we combined each of the eight combinations described above with each of the two sequences of correct responses from prime to probe (repetition vs. change) randomly across two successive mini blocks. We further randomly combined the eight guessing conditions in two successive mini blocks with the two feedbacks (correct vs. wrong).

We further made adjustments in the instructions aiming for fewer exclusions of participants. For one, many participants preferred one of the two response keys for guessing in Experiment 1. So we slightly adapted the advisement in the beginning of the experiment (novel instructions are highlighted in italics) that participants did not need to balance their guesses between the left and right response *within a mini block*. We further presented optional feedback after a block if participants showed a preference for one of the response keys for guessing. We fed back which key they preferred and reminded them to make a spontaneous decision based on their gut feeling, as if flipping a coin. We also presented feedback after a block if rule-based prime responses or probe responses were less than 75% correct to prevent exclusions due to these criteria. We informed participants about which task they did not respond to quickly or accurately enough. If accuracy was low for rule-based prime responses, we reminded them to remember the assignment of pictograms to responses as quickly and accurately as possible. If accuracy was low in the probe task, we encouraged them to concentrate on classifying the color quickly and accurately while trying to ignore the pictogram.

### Results

#### Data treatment

We excluded and replaced two participants for whom the experiment was aborted prematurely because of a technical failure. The first mini block was excluded as practice. We excluded and replaced 15 participants who provided less than 75% correct rule-based prime responses. The remaining participants responded correctly in at least 75% of the probes. Another six participants preferred one of the response keys in more than 70% of the guessed prime responses, and they were therefore excluded and replaced. We excluded prime-probe pairs with an error in the preceding probe (6.8% commission errors and 0.2% omission errors). Afterward, we excluded trials with an early response before the prime during fixation (< 0.1%), trials with a commission error in rule-based primes (4.9%), trials with an omission of the prime response (0.2%) or with a response between prime and probe (0.4%). For the analysis of the percentage of commission errors in the probe, we excluded probe omission errors (0.1%). For the analysis of probe RTs, we further excluded commission errors in the probe (6.7%) and then outlier trials where RTs of the probe deviated more than 2.5 standard deviations from their respective cell mean (1.9%). All remaining participants delivered at least ten observations in all experimental cells after these exclusions and therefore entered the analyses.

#### Data analyses

**Fig. 6:**
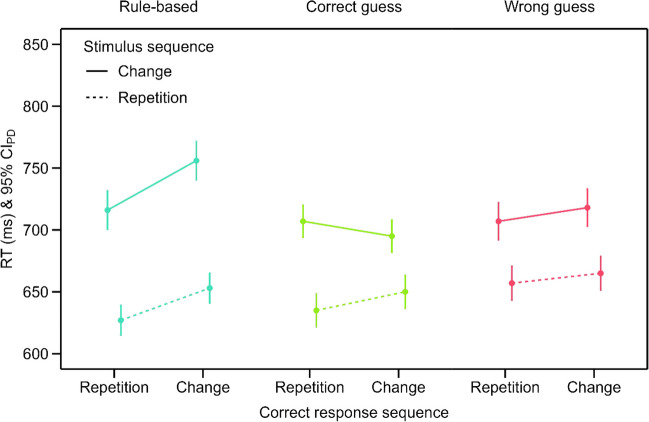
Detailed results for the response time (RT) in Experiment 2. Means are depicted as a function of stimulus sequence and correct response sequence for the three prime types, that is, rule-based, correct guess and wrong guess. Error bars represent the 95% confidence interval of the paired sdifferences between correct response sequences (CI_PD_), computed separately for stimulus sequences and prime types

**Fig. 7:**
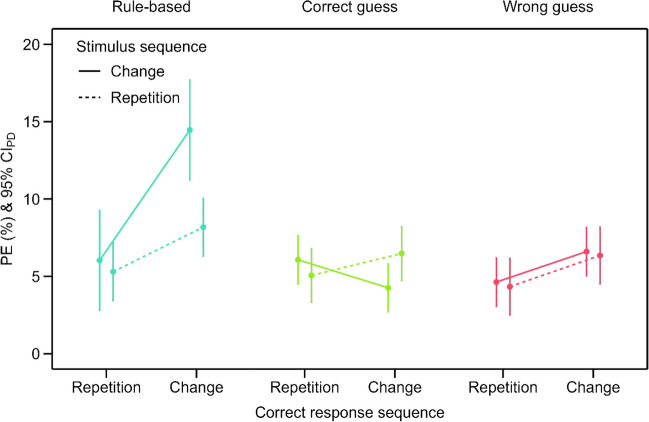
Detailed results for the percentage error (PE) in Experiment 2. Means are depicted as a function of stimulus sequence and correct response sequence for the three prime types, that is, rule-based, correct guess and wrong guess. Error bars represent the 95% confidence interval of the paired differences between correct response sequences (CI_PD_), computed separately for stimulus sequences and prime types

As in Experiment 1, we analyzed RTs of correct probe responses in a 2 × 2 × 3 ANOVA with the within-subject factors prime type (rule-based vs. correct guess vs. wrong guess) × stimulus sequence (repetition vs. change) × correct response sequence (repetition vs. change). We scrutinized significant two-way interactions in two-tailed paired-samples *t*-tests. If the three-way interaction was significant, we also computed sequential stimulus–response effects. We assessed whether these effects (1) differed between prime types in separate one-tailed paired-samples *t*-tests (rule-based > correct guess; rule-based > wrong guess; correct guess > wrong guessed) and (2) whether they were individually larger than 0 in three one-sample *t*-tests. If any of these six tests was not significant, we tested the respective differences for equivalence in two one-tailed samples *t*-tests with the R package TOSTER (Lakens et al., 2018). We tested whether the difference was larger than d_z_ = -0.34 and smaller than d_z_ = 0.34, i.e., we used the effect size that we had reasonable power to detect as test value (see *Participants*). We analyzed percentages of commission errors in the probe as described above for RTs to address potential speed-accuracy trade-offs. Figure 3 shows key results for the RT and error analyses. In the Appendix, we present absolute RTs and percentages of commission errors for each design cell in Figs. 6 and 7 and also in Tables 3 and 4.

#### Response times

The main effect of prime type was significant (see Fig. 6 in the Appendix), *F*(2, 150) = 5.52, *p* = 0.009, η_p_^2^ = 0.07 (ε = 0.80), with similarly slow responses after rule-based responses and wrong guesses but faster responses after correct guesses. Stimulus repetitions were faster than stimulus changes, *F*(1, 75) = 139.42, *p* < 0.001, η_p_^2^ = 0.65, and correct response repetitions were faster than correct response changes,* F*(1, 75) = 22.85, *p* < 0.001, η_p_^2^ = 0.23. Prime type interacted with stimulus sequence, *F*(2, 150) = 26.37, *p* < 0.001, η_p_^2^ = 0.26, indicating that benefits of repeating over changing the stimulus were largest for rule-based primes, *t*(75) = 11.95, *p* < 0.001, *d*_*z*_ = 1.37, followed by correct guesses, *t*(75) = 9.30, *p* < 0.001, *d*_*z*_ = 1.07, and the smallest but still a large effect emerged for wrong guesses, *t*(75) = 7.82, *p* < 0.001, *d*_*z*_ = 0.90. Prime type also interacted significantly with correct response sequence, *F*(2, 150) = 11.12, *p* < 0.001, η_p_^2^ = 0.13 (ε = 0.89), as benefits of correct response repetitions over changes only emerged for rule-based primes, *t*(75) = 6.22, *p* < 0.001, *d*_*z*_ = 0.71, not for correct guesses, |*t*|< 1, or wrong guesses, *t*(75) = 1.89, *p* = 0.063, *d*_*z*_ = 0.22. The two-way interaction between stimulus sequence and correct response sequence was not significant (see Fig. 3), *F* < 1, but the three-way interaction between all three factors was, *F*(2, 150) = 4.92, *p* = 0.009, η_p_^2^ = 0.06.

The sequential stimulus–response effect was not larger for rule-based primes than correct guesses, *t*(75) = -3.30, *p* > 0.999, *d*_*z*_ = -0.38, but also not significantly equivalent |*t*|< 1, 90% CI_*dz*_ = [-0.58, -0.19]. It was also not larger for rule-based primes than wrong guesses, |*t*|< 1, but instead equivalent, *t*(75) = 2.22, *p* = 0.015, 90% CI_*dz*_ = [-0.28, 0.10]. The sequential stimulus–response effect was larger for correct than wrong guesses, *t*(75) = 2.18, *p* = 0.016, *d*_*z*_ = 0.25. It was only significantly positive for correct guesses, *t*(75) = 2.99, *p* = 0.002, *d*_*z*_ = 0.34, not for rule-based primes, *t*(75) = -1.57, *p* = 0.940, *d*_*z*_ = -0.18 (but also not equivalent, *t*(75) = 1.39, *p* = 0.084, 90% CI_*dz*_ = [-0.38, 0.01]), or for wrong guesses, |*t*|< 1 (significantly equivalent, *t*(75) = 2.64, *p* = 0.005, 90% CI_*dz*_ = [-0.23, 0.15]).

#### Percentage of commission errors

The percentage of commission errors differed between prime types (see Fig. 7 in the Appendix), *F*(2, 150) = 30.75, *p* < 0.001, η_p_^2^ = 0.29 (ε = 0.87), with similarly few errors after correct and wrong guesses and more errors after rule-based primes. More errors emerged for stimulus changes than for stimulus repetitions, *F*(1, 75) = 8.72, *p* = 0.004, η_p_^2^ = 0.10, and for correct response changes than for repetitions, *F*(1, 75) = 20.17, *p* < 0.001, η_p_^2^ = 0.21. Prime type modulated the effect of stimulus sequence, *F*(2, 150) = 19.20, *p* < 0.001, η_p_^2^ = 0.20, as the benefit of stimulus repetition over changes only emerged for rule-based primes, *t*(75) = 5.72, *p* < 0.001, *d*_*z*_ = 0.66, not for correct guesses, *t*(75) = -1.15, *p* = 0.255, *d*_*z*_ = -0.13, or wrong guesses, |*t*|< 1. Prime type further interacted with correct response sequence, *F*(2, 150) = 15.93, *p* < 0.001, η_p_^2^ = 0.18 (ε = 0.89), indicating that benefits of correct response repetitions only emerged for rule-based primes, *t*(75) = 5.13, *p* < 0.001, *d*_*z*_ = 0.59, and wrong guesses, *t*(75) = 3.06, *p* = 0.003, *d*_*z*_ = 0.35, but not for correct guesses, |*t*|< 1. The two-way interaction of stimulus and correct response sequence was not significant (see Fig. 3), *F*(1, 75) = 1.04, *p* = 0.311, η_p_^2^ = 0.01, but the three-way interaction between all factors was, *F*(2, 150) = 13.74, *p* < 0.001, η_p_^2^ = 0.15.

The sequential stimulus–response effect was not larger for rule-based primes than correct guesses, *t*(75) = -5.36, *p* > 0.999, *d*_*z*_ = -0.61, but not significantly equivalent, *t*(75) = -2.40, *p* = 0.990, 90% CI_*dz*_ = [-0.84, -0.42]. Similarly, it was not larger for rule-based primes than for wrong guesses, *t*(75) = -2.97, *p* = 0.998, *d*_*z*_ = -0.34, but also not equivalent, *t*(75) = -0.01, *p* = 0.504, 90% CI_*dz*_ = [-0.55, -0.15]. The sequential stimulus–response effect was larger for correct than for wrong guesses, *t*(75) = 2.05, *p* = 0.022, *d*_*z*_ = 0.24. The sequential stimulus–response effect was only significantly positive for correct guesses, *t*(75) = 2.94, *p* = 0.002, *d*_*z*_ = 0.34, not for rule-based primes, *t*(75) = -3.80, *p* > 0.999, *d*_*z*_ = -0.44 (but also not equivalent, *t*(75) = -0.84, *p* = 0.797, 90% CI_*dz*_ = [-0.65, -0.25]), or for wrong guesses, |*t*|< 1 (significantly equivalent, *t*(75) = -2.92, *p* = 0.002, 90% CI_*dz*_ = [-0.19, 0.20]).

#### Exploratory analyses

As in Experiment 1, we analyzed RTs of the prime in a two-tailed paired-samples *t*-test comparing rule-based responses and guesses. We excluded 2.8% of the trials as outliers and included all participants in the analysis because they delivered at least ten observations in each experimental cell. RTs in the prime were again smaller for rule-based responses (*M* = 690 ms) than for guesses (*M* = 782 ms),* t*(75) = 9.73, *p* < 0.001, *d*_*z*_ = 1.12.

### Discussion

Experiment 2 replicated the modulation of correct response sequence by stimulus sequence for correct guesses, suggesting binding and retrieval for these actions. Wrong guesses again did not show any indication of binding and retrieval. Our equivalence tests further demonstrate that binding and retrieval emerges, at best, only rarely or weakly for wrong guesses.

While we did find evidence for binding of stimuli and correctly guessed responses, the data of this second experiment did not yield any indication for binding and retrieval of stimuli and rule-based responses, as shown by a lack of sequential stimulus–response effects for the latter. We think, however, that a fair comparison between rule-based and guessed actions is unfortunately again not possible here. The result pattern of the rule-based trials seems to be largely driven by the perfect predictability of the irrelevant response suggested by the probe pictogram identity. Specifically, already upon encountering the prime pictogram in a rule-based trial, the response (left vs. right) that the probe pictogram would suggest (i.e., the same response) became perfectly predictable. In guessing trials, however, because of stimulus change trials, the possibility remained that the probe pictogram would not suggest any response. Recent evidence points to weaker binding and retrieval effects of irrelevant stimuli and concurrent responses if the identity of the irrelevant stimulus of the probe is perfectly predictable compared to a less predictable probe stimulus (Schmalbrock et al., 2023). Accordingly, the pictogram in the probe might not have retrieved its former response upon repetition from the prime in our study. Instead, the participants might have either kept the rule-based prime response active during the probe or anticipated the probe response, leading to benefits of repeating the response from prime to probe. Speculatively, the reversal of the traditional binding and retrieval pattern with stronger benefits of repeating over changing the response for stimulus changes than stimulus repetition indicates that a change of the pictogram drew more attention than a stimulus repetition. Increased attention to the changed pictogram might then have strengthened retrieval of the rule-based response in addition to the (residual or anticipated) response activation from the prime.

Although we did not find the right operationalization here to study the impact of instructed rules on binding, we hope that future studies will succeed in identifying whether bindings are built from scratch or whether they are constructed on the basis of existing memory traces.

## General discussion

Our initial question was whether the circumstances of how people act – guided by rules or guessing – shape how these situations affect following performance. The current study therefore set out to investigate (1) the impact of instructed rules on binding and retrieval of actions and acted-upon stimuli and (2) binding and retrieval for correct and wrong guesses to stimuli when correctness is only resolved through feedback after the response. From the data, we conclude that binding can emerge in guessing situations, depending on explicit feedback after taking the guess. Feedback that the guess was correct paves the way for binding of the guessed response to the acted-upon stimulus. Feedback that the guess was wrong prevents binding with the guessed response from happening, leads to unbinding of the stimulus and the guessed response, or prevents response retrieval in the probe. So binding for unsuccessful responses clearly differs between situations where rules are available before taking action and binding and retrieval between stimulus and correct response are apparent (e.g., Foerster et al., 2022a; Parmar et al., 2022), and situations where they only become available through feedback following a guess and binding and retrieval of the stimulus and correct response do not show. However, the data do not allow us to infer whether rule-based stimulus–response bindings feed directly into experience-based stimulus–response bindings.

### Accumulation of binding strength

Binding and retrieval occur not only for task-relevant stimuli and their responses but also for coincidental irrelevant stimuli and responses, although these stimuli are not informative for responding successfully (e.g., Frings et al., 2007; Giesen et al., 2012; Moeller & Frings, 2014). However, sequential stimulus–response effects are considerably weaker when the stimuli are irrelevant than relevant (e.g., Frings et al., 2022; Pfister et al., 2022). These differences appear to already arise during binding while decay functions are similar for both stimulus types (Frings et al., 2022). We suggest that the accessibility of stimulus–response rules for relevant but not for irrelevant stimuli feeds into binding strength. The comparison of instructed and guessed responses is unfortunately not informative for the former assumption. However, the small effect sizes of sequential stimulus–response effects for correct guesses (*d*_*z*_ ≤ 0.34) at least hint in that direction because they are in the ballpark of what has been observed for irrelevant stimuli and responses. Having a clearcut demonstration of the accumulation of binding strength via the instruction and the application of task rules would support recent considerations on how such transient bindings might develop into durable memory traces (Frings et al., 2023).

### *Encoding of features*^5^

On the one hand, binding and retrieval might indeed be weaker for guesses because instructed memory traces are not available to kickstart binding from an elevated activation level (Frings et al., 2023). On the other hand, prioritized memorizing of the instructed stimulus–response rules might come at the cost of weaker encoding of the stimulus identities in guessing situations (during the instructions and/or during the guessing trials) and of the guessed responses (during the guessing trials). If these features are not properly encoded in the first place, how would they be bound?

Exploratory analyses show that prime responses were faster if they were rule-based than guesses. One interpretation of this pattern is that participants recognized stimuli for guesses only as not being rule-based in a relatively slow process without encoding the specific identity of these stimuli and their responses. Retrospectively, correct feedback for the guess might have drawn attention to the identity of features, leading to their encoding and therefore allowing for their binding and later retrieval.

However, for all prime types, that is rule-based responses, correct guesses and wrong guesses, we found similar benefits of repeating the stimulus and of repeating the correct response from prime to probe in Experiment 1. These benefits suggest that the identity of stimuli and responses was encoded also for guesses. In Experiment 2, at least stimulus repetition benefits emerged for all prime types in RTs, albeit they were weaker for correct guesses than rule-based responses and they were the weakest but still substantial in size for wrong guesses. In error rates, these benefits were absent for both guesses. Benefits of repeating the correct response emerged significantly for rule-based responses in RTs and percentages of commission errors while a smaller benefit occurred only for wrong guesses in errors. As such, encoding of stimulus and response features might have been weaker but not completely absent for guesses in our paradigm.

A systematic approach to investigating the role of encoding in guessing situations could be a comparison of guesses without a motivation to learn (i.e., as implemented here) as compared to a setting with a motivation to learn the correct response to a stimulus. For example, during the instruction phase, half of the stimuli might be marked to appear again for a later test. That is, participants would know that they will not only guess the correct response to these stimuli once but that they will have to provide the correct response in a subsequent test. Such a procedure should encourage encoding of the identity of stimuli and responses and therefore boost binding and retrieval for guesses, potentially even for wrong guesses.

The fact that rule-based responses were initiated faster than guesses, probably because of stronger encoding of rule-based situations, could also modulate binding via the temporal distance between the onset of the stimulus and the response. With shorter RTs, the response occurs closer to the onset of the stimulus. To the best of our knowledge, there is no investigation in the literature of the impact of the time that passes between (the onset) of a stimulus and a response on binding. However, it seems plausible that binding might be particularly strong when features appear temporally closer to each other. As the stimulus stayed on the screen until a response was registered, they always coincided similarly and independently of the RT in the current paradigm.

### Types of errors and their consequences

So far binding and retrieval has been studied for two extremely different types of unsuccessful actions. On the one hand, we studied errors for situations in which a few stimuli mapped to only a few responses and where these rules were thoroughly instructed and practiced (Foerster et al., 2023, 2021, 2022a; Parmar et al., 2022). On the other hand, the current study targeted situations that did not provide any information about the correct response except for it being one out of two possible responses. Speculatively, correct action plans might have been put into place all along for unsuccessful action episodes in the former rule-based situation. That is, upon stimulus presentation the correct action plan might have been set up but a sudden, unsystematic burst in activation triggered the execution of the incorrect response instead. As such, binding of the correct response might only emerge for errors that occur despite the formation of correct action plans before their execution. A fruitful avenue for exploring this hypothesis could be the comparison of different error types in flanker tasks. In these tasks, irrelevant flanker stimuli provoke errors when participants attend to them too strongly, but participants also commit errors that are not related to the identity of the irrelevant stimuli (e.g., Maier et al., 2008, 2011). Therefore, the former error type should be more likely triggered by an incorrect action plan than the latter error type for which correct action plans might be more prevalent instead. As such, these errors might also differ in terms of whether correct or erroneous aspects of the response are bound.

Intriguingly, the data still point to a heightened activation of the actual correct response as probe responses were more accurate in sequences with a repetition than with a change of the correct response even after wrong guesses. This pattern suggests that wrong guesses came with a typical urge to correct the error (e.g., Crump & Logan, 2013; Rabbitt, 1966, 1978), merely based on feedback after responding. However, the data demonstrate that such an activation of the correct response does not necessarily lead to its binding to the stimulus. In the current setting, we did not provide an external motivator to learn from guessing. Speculatively, providing such a motivator, for example in terms of an incentivized memory test as suggested above, might pave the way for binding between the actual correct response and the stimulus after receiving feedback that a guess was wrong.

### Participants’ degrees of freedom

What did participants make out of the instructions to guess the correct response to half of the stimuli? Above, we discussed the possibility that they might have prioritized encoding and memorizing explicit stimulus–response rules, and therefore might have lost sight of stimuli and responses related to guesses. Our data further show that a considerable proportion of participants preferred one response option over the other, although we explicitly asked them to decide spontaneously between them and fed back biased responding in the second experiment.

Participants might have resorted to other strategies that went undetected like coming up with their own rules for already responding to guessing stimuli during the instructions. In this case, there could be an impact of rules on binding even for guesses. If this strategy had been used largely by our participants, absolute performance should, however, be similar for the different prime types. The same holds true for sequential effects, especially correct guesses and rule-based responses where participants applied instructions (by the experimenter or themselves) successfully in the prime.

Participants might have also resorted to preparing a response beforehand so that they could execute it efficiently whenever they encountered a stimulus that required a guess. For example, they might have used one key to all guessing stimuli of one block and the other key in another block, they might have responded in an alternating pattern to guessing stimuli, etc. Such response preparation could then affect binding. Bindings are already formed when an action is planned, even if the execution of that action is delayed (e.g., Hommel et al., 2001; Mocke et al., 2022; Stoet & Hommel, 1999). As such, prepared “guessing” responses could be bound to stimuli that we did not intend for binding (e.g., the fixation cross), potentially hampering binding of this response to the pictogram. As participants could not predict whether the upcoming prime would require a rule-based response or a guess in most trials,^6^ such strategies for response preparation might have brought noise to all types of primes.

## Conclusion

Does feedback affect the ubiquitous process of stimulus–response binding and retrieval? The present experiments provide strong evidence that this is indeed the case in guessing situations, where positive feedback promoted binding and retrieval whereas negative feedback prevented binding and/or retrieval. This pattern stands in stark contrast to rule-based actions, where previous research did not observe an impact of feedback. Comparing both patterns further hints at a modulating impact of rules on binding and retrieval of performed actions, even though the current study cannot deliver direct evidence for such an interplay. Additional experimental work in the present approach, however, might reveal when bindings are built from scratch and when their creation is rooted in existing instructed memory traces.

## Data Availability

At the Open Science Framework (10.17605/OSF.IO/7YCU8) everyone has access to the preregistrations (Experiment 1: 10.17605/OSF.IO/DCRFJ and Experiment 2: 10.17605/OSF.IO/84MU3), experimental programs with stimuli, as well as the data and the analysis code for both experiments.

## Appendix

**Table 1:** Descriptive statistics for the response times in Experiment 1

Prime type	Stimulus sequence	Correct response sequence	*M*	*SD*
Rule-based	Repetition	Repetition	616	139
		Change	659	130
	Change	Repetition	680	158
		Change	666	149
Correct guess	Repetition	Repetition	620	148
		Change	641	130
	Change	Repetition	666	149
		Change	661	156
Wrong guess	Repetition	Repetition	632	127
		Change	640	145
	Change	Repetition	681	152
		Change	670	144

**Table 2:** Descriptive statistics for the percentage of errors in Experiment 1

Prime type	Stimulus sequence	Correct response sequence	*M*	*SD*
Rule-based	Repetition	Repetition	3.86	5.06
		Change	9.56	6.40
	Change	Repetition	7.05	8.70
		Change	3.65	4.55
Correct guess	Repetition	Repetition	4.37	6.87
		Change	4.50	5.66
	Change	Repetition	4.11	5.53
		Change	3.25	4.09
Wrong guess	Repetition	Repetition	4.00	4.03
		Change	6.76	8.06
	Change	Repetition	3.46	4.80
		Change	6.29	9.14

**Table 3:** Descriptive statistics for the response times for Experiment 2

Prime type	Stimulus sequence	Correct response sequence	*M*	*SD*
Rule-based	Repetition	Repetition	627	121
		Change	653	104
	Change	Repetition	716	153
		Change	756	165
Correct guess	Repetition	Repetition	635	115
		Change	650	107
	Change	Repetition	707	135
		Change	695	135
Wrong guess	Repetition	Repetition	657	116
		Change	665	119
	Change	Repetition	707	140
		Change	718	135

**Table 4:** Descriptive statistics for the percentage of errors in Experiment 2

Prime type	Stimulus sequence	Correct response sequence	*M*	*SD*
Rule-based	Repetition	Repetition	5.30	5.92
		Change	8.18	7.31
	Change	Repetition	6.03	6.24
		Change	14.46	13.01
Correct guess	Repetition	Repetition	5.06	5.71
		Change	6.48	6.58
	Change	Repetition	6.07	6.10
		Change	4.26	5.02
Wrong guess	Repetition	Repetition	4.33	5.56
		Change	6.35	6.85
	Change	Repetition	4.63	5.61
		Change	6.60	5.59

## Footnotes

1 Note that we have the same power calculations in the preregistration but incorrectly wrote “to detect the smaller effect size,” which should instead read “to detect a medium-sized effect of *d*_*z*_ = 0.50.” Importantly, we collected the number of analyzable datasets as preregistered.

2 In the preregistration, we announced that we would also assess sequential stimulus–response effects in an ANOVA with the single factor of prime response type. However, the results of this ANOVA would have been redundant with a three-way interaction of the full ANOVA, so we therefore decided to skip it.

3 As instructions for rule-based responses always map the same correct response to a prime-probe pair, there should be a general benefit of repeating compared to changing the correct response for these prime types. This compatibility effect between activated responses can only emerge for rule-based responses, not for guesses (predicting a two-way interaction between prime type × correct response sequence). This pattern did indeed emerge in the data. We thank an anonymous reviewer of a previous version of the manuscript for directing us to this aspect of the paradigm.

4 The effect sizes we report here differ slightly from the ones reported in the preregistration because there was an error in the analysis syntax of Experiment 1 when we preregistered Experiment 2. Note that the selection of detectable effects, d_z_ ≥ 0.34, matches the preregistration.

5 We thank Jan De Houwer for motivating this discussion and for suggesting suitable exploratory analyses.

6 At the end of a mini block, such a prediction is possible in principle if participants keep track of which pictograms already appeared.

## Code availability

See *Availability of data and materials*.
